# Prevalence and associated factors of pneumonia among under-fives with acute respiratory symptoms: a cross sectional study at a Teaching Hospital in Bushenyi District, Western Uganda

**DOI:** 10.4314/ahs.v21i4.25

**Published:** 2021-12

**Authors:** Gloria Kiconco, Munanura Turyasiima, Andrew Ndamira, Ortiz Arias Yamile, Walufu Ivan Egesa, Martin Ndiwimana, Melvis Bernis Maren

**Affiliations:** 1 Department of Pediatrics, Kampala International University School of Health Sciences, Western Campus; 2 African Medical and Research Foundation (AMREF) Africa, Mbarara, Uganda

**Keywords:** Pneumonia, prevalence, under-fives

## Abstract

**Objectives:**

This study assessed the prevalence and associated factors of pneumonia among children under-five years presenting with acute respiratory symptoms.

**Methodology:**

This was a cross sectional study at the Pediatric Department of Kampala International University – Teaching Hospital, from the month of April to August 2019. The study included 336 children aged 2 to 59 months presenting with acute respiratory symptoms to the pediatric clinic. Pneumonia diagnosis was made according to the World Health Organization definition, modified by a chest radiograph. Structured questionnaires were used to collect data on socio-demographic, environmental and nutrition factors and multivariate logistic regression analysis using STATA version 13.0 was done to assess for the factors independently associated with pneumonia.

**Results:**

Of the 336 children with acute respiratory symptoms, eighty-six, 86 (25.6%) had pneumonia. Factors significantly associated with pneumonia included: age below 6 months (OR=3.2, 95%CI=1.17–8.51, p=0.023), rural residence (OR=5.7, 95%CI=2.97–11.05, p <0.001), not up-to-date for age immunization status (OR=2.9, 95%CI=1.05–7.98, p=0.039), severe acute malnutrition (OR=10.8, 95%CI=2.01–58.41, p=0.006), lack of exclusive breastfeeding during the first six months (OR=2.9, 95%CI=1.53–5.53, p=0.001) and exposure to cigarette smoke (OR=3.0, 95%CI=1.35–6.80, p=0.007).

**Conclusion:**

The prevalence of pneumonia in children under-five years was high. Most of the factors associated with pneumonia are modifiable; addressing these factors could reduce this prevalence.

## Introduction

Pneumonia is the most common infectious cause of death in children accounting for 16% of all deaths of under-fives worldwide, it is most prevalent in south Asia and sub-Saharan Africa 1 and is the second leading cause of under-five in-patient mortality in Uganda 2. Systematic review and meta-analysis of data on pneumonia across East African countries, estimated the average prevalence of pneumonia in under-fives at 34% 3. In Uganda, 80% of children under five years that sought treatment from a health worker had symptoms of an acute respiratory infection 4. A study was done at Mulago national Referral Hospital in Uganda and recorded a prevalence at 53.7% 5. Studies in different African countries have estimated pneumonia prevalence among under-fives between 16% and 33% 6, 7, 8.

The leading risk factors that contribute to pneumonia incidence are lack of exclusive breastfeeding, under-nutrition, indoor air pollution, low birth weight, over crowding lack of immunization and comorbid conditions^9^,^10^. Children with compromised immune systems like malnutrition especially in infants not exclusively breastfed are at higher risk of developing pneumonia^1^, ^9^. The burden of child hood Pneumonia has declined but the rate of decline is slow compared to other infectious diseases in children 1.

The government of Uganda has implemented the strategies adapted from Global Action Plan for Prevention and Control of Pneumonia (GAPP) which includes a combination of interventions to protect, prevent, and to treat pneumonia for example vaccination, encouraging exclusive breastfeeding for six months and access to proper pneumonia treatment, however in Uganda we are still below the national targets 4, 11, 12 which has contributed to a high Under-five mortality 4. This study determined the prevalence of pneumonia and described factors associated with pneumonia among children between 2 and 59 months with acute respiratory symptoms attending to Kampala International Teaching Hospital (KIU-TH) in Bushenyi district Western Uganda.

## Methods and materials

### Study design and participants

This study was a hospital based descriptive cross sectional and analytical study in the Pediatric Department of Kampala International University Teaching Hospital (KIU-TH), a private tertiary hospital located in Ishaka-Bushenyi municipality Bushenyi district Western Uganda. It serves as a referral center for hospitals and health centers around the districts of Bushenyi, Sheema, Buhweju and Mitooma. KIU-TH provides general and specialized services, has outpatient and inpatient departments for each discipline with an emergency wing and intensive care unit. The Pediatric department has five 5 sections: pediatric ward, neonatal ward, Pediatric OPD and emergency. The study was conducted in the pediatric ward, pediatric OPD and emergency ward. According to KIU T-H records in 2018, approximately 280 children under five years attend to KIU T-H per month and around 70% present with acute respiratory symptoms and about one child died of pneumonia every month.

Three hundred thirty-six (336) children aged between 2 and 59 months presenting with acute respiratory symptoms at the pediatric out patient, emergency and pediatric ward were consecutively recruited into the study from the months of April to August 2019.

### Study procedure

All the children aged 2–59 months who attended to KIU-TH with acute respiratory symptoms (running nose, cough, fast breathing and/or difficulty in breathing) during the study period were consecutively recruited for the study. Children with acute respiratory symptoms were identified from the presenting complaint at outpatient clinic, Emergency ward or in the pediatric ward. Consent ws obtained from the children's parents/caretakers after the primary reason for seeking health care had been taken care of and any emergency treatment (if required) given.

We excluded children with obvious clinical features of acute aspiration (near drowning and acute foreign body inhalation) since there was a known cause.

A structured questionnaire was used to capture comprehensive data on acute respiratory symptoms, sociodemographic information, breast feeding history, birth weight, immunization status, environmental factors and comorbid medical conditions like Human Immune Virus infection (HIV), cerebral palsy and heart disease that could be associated with pneumonia. Birth weight was checked from the immunization cards and by direct interview of parents/caretakers for those who did not have the cards at the time of data collection. Urban residence was referred to as cities, municipalities and towns with a population over 2,000 persons 13. Parental smoking as an environmental risk factor for pneumonia applied only when the smoker stays in the same environment with the child.

Physical examination was done for every child to document nutritional status and clinical features of pneumonia including: increased respiratory rate (according to age), chest in drawing and crackles or added sounds on chest auscultation. Chest radiographs were only done for children with clinical features of pneumonia based on World Health Organization Cough and/or difficulty in breathing with fast breathing and/or chest in drawing).

### Pneumonia definition

In this study pneumonia was defined as presence Cough and/or difficulty in breathing with fast breathing and/or chest in drawing 14. These symptoms also present in other many acute respiratory conditions. Therefore the definition was modified with presence of positive chest X-ray findings of pneumonia which included one of the following: infiltrates, consolidation, pleural effusion, and empyema indicated pneumonia^15^

However, children who had clinical features of pneumonia using WHO/IMCI criteria but with normal chest X-ray and had no other identified respiratory focus of infection like pharyngitis, tonsillitis, and otitis media were considered to have pneumonia. This was because a normal chest X-ray might not rule out pneumonia since sensitivity is around 83.3% 16. Children with identified respiratory focus of infection and had no features of pneumonia on chest Radiograph were given other respective diagnoses according to the examination findings

### Severe pneumonia

Was defined as presence of pneumonia plus one or more of the danger signs (lethargy, dehydration, cyanosis, vomiting everything, failure to feed, convulsions, oxygen saturation <90%, axillary temperature > 39.5degrees centigrade, severe chest in-drawing) 14.

### Nutritional status

Nutritional status was assessed according to the WHO nutritional charts 17 and classified as normal nutritional status, moderate acute malnutrition (MAM) and severe acute malnutrition (SAM).

Weight was measured using a weighing scale (salter type baby weighing scale for infants and those who could not stand) and digital electric weighing scale when the child completely undressed and rounded to the nearest 0.1kg. The body length/height was measured using a Stadiometer and length measuring board and recorded in centimeters, Mid-upper arm circumference (MUAC) was measured using a MUAC tape and recorded in centimeters.

### Immunization status

Child health cards were used to assess the immunization status of children. Appropriate history about immunization using a checklist was used for caretakers who did not have immunization cards at the time of data collection. Children who had not received all vaccinations expected for their age or had never been immunized at all were labeled not up to-date for age.

### Data management

Data collection tools were pretested to ensure reliability and validity, questionnaires were translated into local language (Runyankole). Legibility was ensured by daily auditing of the questionnaires. Chest x-rays were taken by a radiologist and interpreted properly. Radiographic films were attached to the respective questionnaires to avoid misplacement. Privacy and confidentiality were ensured by conducting interviews in the closed clinic and all data collected kept in a lock and password protected computer. After collection, data was arranged, coded and entered into the computer using the EXCEL 2016 then imported to STATA version 13.0 (Statacorp, College station, USA) for analysis.

### Data analysis

Data was analyzed using STATA version 13.0 (Statacorp, College station, USA). The prevalence of pneumonia among children presenting with acute respiratory symptoms was analyzed using frequencies, percentages and corresponding 95% confidence interval (CI). Factors associated with pneumonia were analyzed using univariate and multivariate logistic regression. Factors with p-value ≤0.2 at univariate analysis qualified to be taken to multivariate analysis. Measures of effect were reported using odds ratios for both crude and adjusted analysis, followed by corresponding 95% CI and p-value. At multivariate analysis, factors with p-value ≤0.05 were considered statistically significant. The results were presented in tables.

### Ethical considerations

Ethical approval was obtained from the research ethics committee (REC) of Kampala International University (Nr UG-REC-023/201902). Informed consent was sought from parents/caretakers of children and the purpose of the study well explained before administering the questionnaire.

### Study limitations

Some parents/caretakers had no immunization cards at the time of recruitment in to the study and were not clear with the information regarding their immunization status and birth weight, however, history on age and site at immunization based on the UNEPI guidelines was used to help them recall though a few of them could not recall at all.

## Results

### Participants' socio-demographics characteristics

Among the 336 children aged 2 to 59 months presenting with acute respiratory symptoms; majority were of age category 24–59 months, males were slightly more than females. Most children were of rural residence and their parents/caretakers had attained primary and secondary level of education. This is shown in table 1.

**Table 1:** Participants' socio-demographic characteristics

Characteristic	Frequency, n (%)
**Age (months)**	
**2–5**	54 (16.1)
**6–11**	69 (20.5)
**12–23**	39 (11.6)
**24–59**	174 (51.8)
**Sex**	
**Male**	179 (53.3)
**Female**	157 (46.7)
**Residence**	
**Urban**	154 (45.8)
**Rural**	182 (54.2)
**Parental/care taker's education**	
**No education**	21 (6.3)
**Primary**	141 (42.0)
**Secondary**	99 (29.5)
**Tertiary**	75 (22.3)
**Marital status**	
**Married**	304 (90.5)
**Single**	32 (9.5)
**Religion**	
**Catholic**	143 (42.6)
**Anglican**	127 (37.8)
**Muslim**	32 (9.5)
**Pentecostal**	34 (10.1)
**Tribe**	
**Banyonkole/Bakiga**	308 (91.7)
**Baganda**	17 (5.1)
**Others**	11 (3.3)

### Participants' medical and environmental characteristics

Majority of the children had their immunization up-to-date for their age, were exclusively breastfed up to six months, had normal nutritional status, with no comorbidity and had normal weight at birth. Most of the children came from families that cook from outside the main house using biomass and their parents/caretakers were non-smokers (Table 2).

**Table 2:** Participant's medical and environmental characteristics

Characteristic	Frequency, n (%)
**Medical characteristics**	
**Immunization status**	
**Up-to-date for age**	309 (92.0)
**Not up-to-date**	27 (8.0)
**Nutritional status**	
**Normal nutrition**	238 (95.2)
**Moderate acute malnutrition**	10 (4.0)
**Severe acute malnutrition**	2 (0.8)
**Exclusive breast feeding for 6 months**	
**No**	71 (21.1)
**Yes**	265 (78.9)
**Comorbidity**	
**No**	327 (97.3)
**Yes**	9 (2.7)
**Birth weight**	
**2.5–3.99**	251 (74.7)
**<2.5**	15 (4.5)
**4+**	27 (8.0)
**Unknown**	43 (12.8)
**Environmental Characteristics**	
**Place of cooking**	
**Indoor**	112 (33.3)
**Outdoor**	224 (66.7)
**Parental/care taker smoking**	
**No**	301 (89.6)
**Yes**	35 (10.4)

### Prevalence of pneumonia among children aged 2 to 59 months presenting with acute respiratory symptoms

Eighty-six (25.6%) children under-five years had pneumonia; 24 (27.9%) of these had the severe form. Pneumonia was more prevalent in children below 6 months of age and affected more of males than females (Table 3).

**Table 3:** Prevalence of pneumonia by age and sex

Prevalence	N	n	%	p-value
**Overall**	336	86	25.6	
**Age specific**				0.127
**2–5**	54	20	37.0	
**6–11**	69	13	18.8	
**12–23**	39	11	28.2	
**24–59**	174	42	24.1	
**Gender specific**				0.767
**Male**	179	47	26.3	
**Female**	157	39	24.8	

### Factors associated with pneumonia among children aged 2 to 59 months presenting with acute respiratory symptoms

Age of the child, place of residence, parental education, religion, parental smoking, immunization status, nutritional status, exclusive breast feeding and having comorbidity were associated with pneumonia at univariate analysis. After adjusting for confounders at multivariate analysis; children who were aged below 6 months, not immunized up-to-date for age, severely malnourished, not exclusively breastfed and exposed to cigarette smoke were more likely to suffer from pneumonia (Table 4).

**Table 4:** Results of bivariate and multivariate analysis for factors associated with pneumonia

Variable	No pneumonia n (%)	Pneumonia n (%)	Unadjusted OR (95% CI)	Adjusted OR (95% CI)	p-value
**Age (months)**					
2–5	34 (13.6)	20 (23.3)	2.5 (1.12–5.74)	3.2 (1.17–8.51)	0.023
6–11	56 (22.4)	13 (15.1)	1.0	1.0	-
12–23	28 (11.2)	11 (12.8)	1.7 (0.67–4.26)	3.0 (0.96–9.54)	0.058
24–59	132 (52.8)	42 (48.8)	1.4 (0.68–2.75)	2.2 (0.91–5.17)	0.081
**Sex**					
Male	132 (52.8)	47 (54.6)	1.0		
Female	118 (47.2)	39 (45.4)	0.9 (0.57–1.52)		
**Residence**					
Urban	139 (55.6)	15 (17.4)	1.0	1.0	-
Rural	111 (44.44)	71 (82.6)	5.9 (3.22–10.91)	5.7 (2.97–11.05)	<0.001
**Parental/caretaker education**					
No education	12 (4.8)	9 (10.5)	4.4 (1.49–12.79)		
Primary	98 (39.2)	43 (50.0)	2.6 (1.23–5.32)		
Secondary	76 (30.4)	23 (26.7)	1.8 (0.80–3.89)		
Tertiary	64 (25.6)	11 (12.8)	1.0		
**Marital status**					
Married	227 (90.8)	77 (89.5)	1.0		
Single	23 (9.2)	9 (10.5)	1.2 (0.51–2.60)		
**Religion**					
Catholic	110 (44.0)	33 (38.4)	1.0		
Anglican	95 (38.0)	32 (37.2)	1.1 (0.64–1.96)		
Muslim	28 (11.2)	4 (4.7)	0.5 (1.56–1.46)		
Pentecostal	17 (6.8)	17 (19.8)	3.3 (1.53–7.25)		
**Tribe**					
Banyankole/B	228 (91.2)	80 (93.0)	1.0		
akiga	13 (5.2)	4 (4.7)	0.9 (0.28–2.77)		
Baganda	9 (3.6)	2 (2.3)	0.6 (0.13–2.99)		
Others					
**Place of cooking**					
Indoor	82 (32.8)	30 (34.9)	1.0		
Outdoor	168 (67.2)	56 (65.1)	0.9 (0.54–1.53)		
**Parental/caretaker** **smoking**					
No	231 (92.4)	70 (81.4)	1.0	1.0	-
Yes	19 (7.6)	16 (18.6)	2.8 (1.36–5.69)	3.0 (1.35–6.80)	0.007
**Immunization status**					
Up-to-date for age	235 (94.0)	74 (86.0)	1.0	1.0	-
Not up-to- date for age	15 (6.0)	12 (14.0)	2.5 (1.14–5.67)	2.9 (1.05–7.98)	0.039
**Exclusive breast** **feeding**					
No	41 (16.4)	30 (34.9)	2.7 (1.57–4.76)	2.9 (1.53–5.53)	0.001
Yes	209 (83.6)	56 (65.1)	1.0	1.0	-
**Nutritional status**					
Normal nutrition	238 (95.2)	70 (81.4)	1.0	1.0	-
MAM	10 (4.0)	8 (9.3)	2.7 (1.03–7.15)	2.9 (0.99–8.64)	0.052
SAM	2 (0.8)	8 (9.3)	13.6 (2.82–65.52)	10.8 (2.01–58.41)	0.006

MAM: moderate acute malnutrition, SAM: severe acute malnutrition

## Discussion

The prevalence of pneumonia was at 25.6% in this study. This prevalence is low compared to findings in a study at Mulago National Referral Hospital Uganda which recorded prevalence of pneumonia in under-fives at 53.7% 5. This could be because of the difference in the study setting. Since Mulago Hospital is near the city, children are likely to be affected by environmental pollution, overcrowding and exposure to smoke due to indoor cooking with biomass which predispose to pneumonia with a high odds of above 1.5 as found in some studies 3, 7,18. Because most children under-five years visit hospitals due to symptoms of acute respiratory infection 3, the prevalence of pneumonia in this study is almost similar to the hospital based studies among all under-fives (33.5%) in Ethiopia 7, 20.2%) in Sudan 19 and (21%) in Kenya 20.

Children of rural residence had 5.7 higher odd of having pneumonia in this study compared to the urban residence, findings are comparable with a study in Ethiopia 6 that reported 4.5 higher odds of developing pneumonia among children of rural residence. The difference could be explained by the low socioeconomic status, low education level in rural areas that are associated with an increased risk of pneumonia 21. However, some studies found an opposite with an increased risk of pneumonia in urban than rural 19 with more children in urban setting suffering from acute respiratory infections compared to those from rural setting 21. This is because of exposure to factors like environmental air pollution and overcrowding in urban areas which have been found to be associated with pneumonia 7,16.

Children aged 2–6 months had 3.2 times higher odd of suffering from pneumonia compared to older infants. A study in Ethiopia 7 found that children who were aged 2–12 month were 2.5 times more likely to develop pneumonia as compared to children above 12 months. The findings can be attributed to the weak immune system in these young infants that allows progression of upper respiratory infection to the lungs causing pneumonia 8.

Children not exclusively breastfeed for six months, not up to date immunized for their age according to national guidelines had almost 3 times odds of having pneumonia compared to the exclusively breast fed and immunized. The findings were comparable with what was found in a study in Brazil 22 where the odds of having pneumonia was 2.4 times higher in children not exclusively breast fed and 2.5 times among those who lacked immunization compared to their counterparts. Exclusive breast feeding and Immunization protects and prevents children from pneumonia 1, this explains why children who lacked these factors had a high chance of developing pneumonia compared to their counterparts. Children with severe acute malnutrition in this study were 11 times more likely to have pneumonia than children with good nutritional status. The findings were similar to related studies in southern Ethiopia 23, in district hospitals of Malawi 24 and in a tertiary Care Centre in Pradesh India 25. This is because malnutrition weakens the immune system and increases the susceptibility of children to acquire pneumonia 9

Having a co-morbidity for example HIV, asthma, cerebral palsy and congenital heart disease was associated with an increased odd of having pneumonia at bivariate analysis. A case control study in eastern Kenya 26 found 3.8 odd of having pneumonia in children with a comorbid condition. These comorbidities compromise the immunity of children rendering them susceptible to developing pneumonia following an upper respiratory tract infection. The results at multivariate analysis did not show any statistical significance and this is because the number of children with co-morbidities in this study was small.

Children who were exposed to cigarette smoke were found to have 3 times higher odds of acquiring pneumonia compared to children who stayed in environment free of cigarette smoke. These findings were comparable to the study in Ethiopia 7 which found that exposure to cigarette smoke increased the odds of having pneumonia by 2.8 times. This is because smoke from the cigarette damages the epithelial lining of the respiratory tract and weakens the innate immune system which allows easy colonization by the microorganisms. However, another study in Ethiopia 6 did not find association between cigarette smoke exposure and pneumonia in children under-five years.

## Conclusion

The prevalence of pneumonia among children presenting with acute respiratory symptoms is high at KIU-TH in Bushenyi and most of the factors associated with pneumonia are modifiable and can be prevented. Therefore emphasis should be put on Health Education to sensitize the community about the preventive measures in addition to appropriate antibiotic treatment of children who already have pneumonia. This is achievable when there is District Health Team Support Supervision to the lower health facilities and continued sensitisation on timely routine immunisation of children, avoiding exposure to cigarette smoke, exclusive breastfeeding, good nutrition practices and prompt antibiotic treatment of children diagnosed with pneumonia

## Figures and Tables

**Fig 1:**
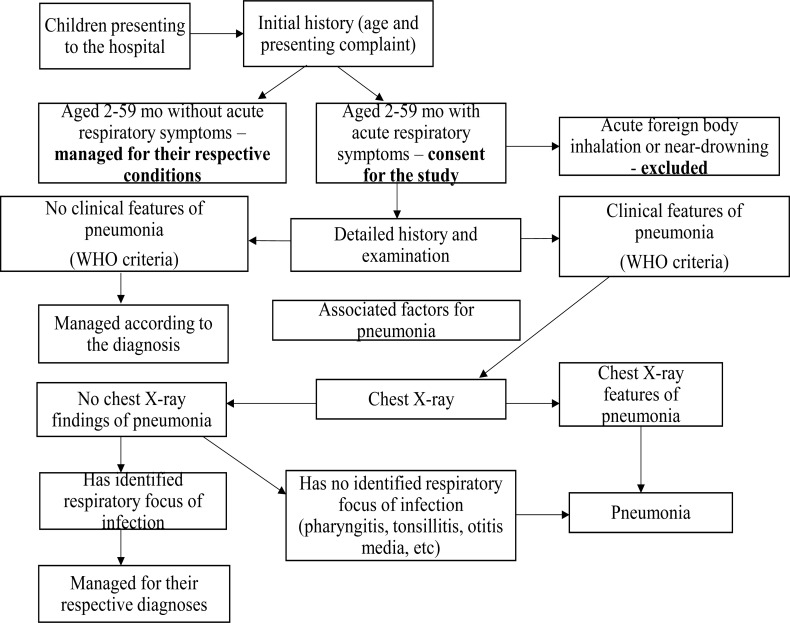
Flow chart showing summery of the study procedure
